# Protective Effects of Milk-Derived Extracellular Vesicles on Colitic Mice via ceRNA Network Involving lncRNAs and circRNAs

**DOI:** 10.3390/foods15091469

**Published:** 2026-04-22

**Authors:** Chunmei Du, Xiaojing Li, Zhaoming Ou, Jin Hu, Suyu Quan

**Affiliations:** 1 College of Coastal Agricultural Sciences, Guangdong Ocean University, Zhanjiang 524088, China; duchunmeim@163.com (C.D.);; 2 Tianjin Key Laboratory of Agricultural Animal Breeding and Healthy Husbandry, College of Animal Science and Veterinary Medicine, Tianjin Agricultural University, Tianjin 300392, China

**Keywords:** circRNA, lncRNA, inflammatory bowel disease, milk, extracellular vesicles

## Abstract

Our previous work demonstrated that bovine milk-derived extracellular vesicles (mEVs) could alleviate the inflammatory response of mice colitis, along with hundreds of differentially expressed (DE) mRNAs. This study further analyzed the profiles of non-coding RNAs (ncRNAs) and explored the correlation with DE mRNAs by constructing ceRNA networks. Six-week-old male C57BL/6 mice were fed either a control diet or a diet added with mEVs for 30 days. Then the mice were given dextran sulphate sodium in drinking water for 7 days to induce colitis. A total of 40 miRNAs, 541 lncRNAs and 643 circRNAs exhibited changes in mEVs pretreatment group. Among these DE miRNAs, mEVs pretreatment significantly increased the expressions of miR-122, miR-147, miR-210, miR-1224, miR-148a, and miR-212, which might participate in the inflammatory response of the colitis models. The expression of Tug1 increased after mEVs pretreatment, while Snhg5 and H19 decreased, which might be involved in intestinal barrier restoration. Functional analysis of the DE ncRNAs suggested mEVs might exert protective effects not only through modulation of inflammatory responses but also by enhancing intestinal stem cell function and epithelial regeneration, which were mainly regulated by Wnt and Hippo signaling pathways according to the ceRNA networks.

## 1. Introduction

Inflammatory bowel diseases (IBD) encompass a group of chronic inflammatory disorders that impact the gastrointestinal tract, including two primary forms: Crohn’s disease and ulcerative colitis [1]. The primary clinical symptoms of IBD include weight loss, repeated strong abdominal pain, diarrhea and bloody stools, affecting an estimated 8.6 million people worldwide [2]. Moreover, IBD are lifelong conditions that can affect individuals of all age groups, with young adults being particularly at high risk [3]. Despite the numerous evidence reporting the involvement of susceptibility genes, gut microbiome, environmental factors, and immune dysregulation in the pathophysiology of IBD [4], recent studies discovered the unexpected importance of intestinal stem cells (ISCs) [5]. The healing of IBD-induced mucosal injury depends on the continuous repair and renewal of intestinal epithelial cells, which are controlled by ISCs within the crypts [6].

Increasing evidence has elucidated the roles of numerous non-coding RNAs (ncRNAs) in the pathogenesis of IBD. About 240 risk gene loci have been identified in IBD patients, with most of these loci are located outside of protein coding regions and appearing to influence ncRNAs [7]. ncRNAs have emerged as important gene regulators at both the transcriptional and translational levels, and they are linked to the pathobiology of IBD [8]. ncRNAs are primarily composed of miRNAs, lncRNAs, and circRNAs. Among them, miRNAs have been the most comprehensively studied, and significant differences in miRNAs expression profiles have been detected in IBD [9]. Furthermore, miRNAs have been reported to influence the inflammatory pathways in IBD, such as regulation of cytokines and chemokines, disruption of autophagy, intestinal epithelial permeability, and activation of nuclear factor-kappa B (NF-κB) [10]. Recent studies demonstrate that lncRNAs might play an important role in inflammatory cascades. For example, lncRNA Neat1 and lncRNA H19 were found to be over-expressed in colitis mice, and they could regulate intestinal epithelial barrier function in IBD pathogenesis [11,12]. In addition, circRNAs, the type of endogenous ncRNAs, could act as miRNAs sponges and also participate in the nosogenesis of IBD. Therefore, characterizing the expression profiles of circRNAs and exploring their function is beneficial for understanding the fundamental molecular mechanisms in IBD [13]. Ye et al. reported that circRNA-103516 was positively associated with pro-inflammatory cytokines and negatively associated with anti-inflammatory cytokines [14]. Taken together, ncRNAs play a critical regulatory role in the pathogenesis of IBD.

Milk, being the primary source of important nutrients and bioactive components that contribute to the development of newborns, also serves as a reservoir of extracellular vesicles (EVs) with the capacity to regulate various biological processes. EVs are membrane surrounded vesicles that are secreted from cells into biofluids, and can carry DNA, RNA, proteins, lipids, and metabolites [15,16]. Our previous study found that bovine milk extracellular vesicles (mEVs) encapsulate a variety of immune-related miRNAs, which may serve as key regulators mediating physiological and pathological functions [17]. Metabolite profiles revealed that most metabolites of mEVs belonged to the category of amino acids, energy metabolites, carbohydrates, fatty acids and associated metabolites [18]. Wolf et al. proved that mEVs could be taken up by intestinal cells by labeling mEVs with the FM4-64 fluorescent dye and measuring cellular fluorescence intensity [19]. Similarly, dextran-fluorescein isothiocyanate-label method showed that bovine milk-derived EVs (mEVs) could survive the harsh gastrointestinal tract environment and reach the intestine to exert their biological functions [20]. Hence, it is reported that mEVs could alleviate ulcerative colitis through the above cargo [21].

Our group found that mEVs could change the serum concentrations of lipids and amino acids which are beneficial for anti-inflammation, as well as the gut microbial community structure in mice [22]. In addition, we proved the protective roles of mEVs on mice chronic colitis models by various means such as disease activity index, length of colon tissues, histological sections of colons, blood metabolite concentrations and gut microbiota [23]. We also found mEVs could alleviate mice acute colitis in many ways, especially through regulating the gene expression of some pro-inflammatory cytokines, chemokine ligands and chemokine receptors [24,25]. However, it remains unknown that whether or not the changed gene expressions were regulated by ncRNAs in colitis during this process. So, this study further explored the expression profiling of miRNAs, lncRNAs and circRNAs of mice with colitis in response to mEVs, to elucidate the underlying protective mechanism by constructing ceRNA network.

## 2. Materials and Methods

### 2.1. Isolation and Characterization of mEVs

The Raw milk was centrifuged at 3000× *g* for 30 min at 4 °C, and followed by 12,000× *g* at 4 °C for 60 min to remove fat, cells, and large debris. The supernatant was centrifuged at 30,000× *g* and 75,000× *g* for 60 min separately, and followed by ultracentrifugation at 120,000× *g* for 90 min (XPN-100; Beckman, Danvers, MA, USA) to obtain EVs. Then, the isolated EVs were filtered through 0.22 μm filter membrane (Millipore, Billerica, MA, USA).

**Figure A1:**
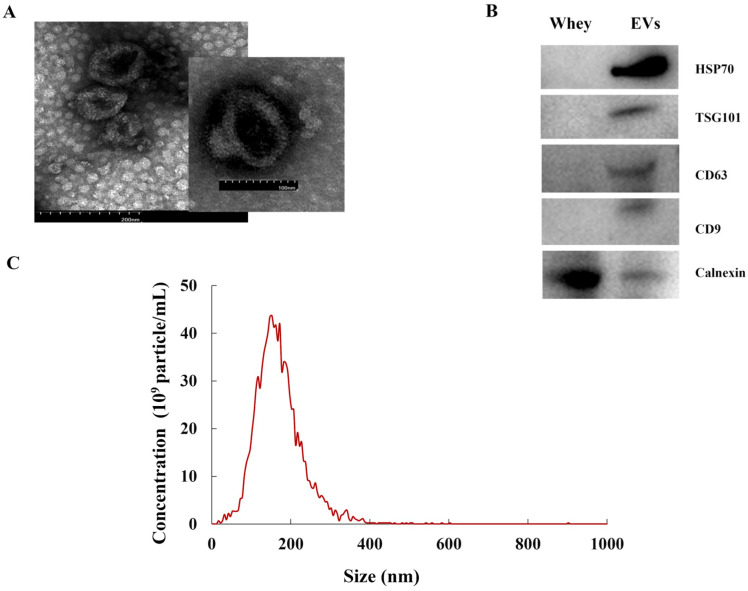
Results of the morphology (**A**), particle size (**B**), and surface markers (**C**) in mEVs.

The morphology of mEVs was examined by transmission electron microscopy (TEM) (HT7700, Hitachi, Tokyo, Japan). The size distribution and particle concentrations of mEVs fractions were determined using nanoparticle tracking analysis (NTA) with Zeta View PMX 110 (Particle Metrix, Meerbusch, Germany). Finally, Western blotting was performed to detect protein markers HSP70, TSG101, CD63, and CD9 (ab275018, Abcam, Cambridge, UK) in mEVs fractions. Calnexin (ab275018, Abcam, UK), as the negative marker protein, was also identified. Automatic, image analysis system (Tanon-4800, Shanghai, China) was used to visualize the proteins. The results of morphology and surface markers are presented in Figure A1.

### 2.2. Animals and Treatment

The animal experiments were approved by Animal Ethics Committee of Chinese Academy of Agricultural Sciences (approval number: IAS2021-235). Twenty-four SPF male C57BL/6 were used. Mice had free access to diet and water. Colitis was induced by administration of dextran sulfate sodium (DSS) (36,000–50,000 kDa; MP Biomedicals, Santa Ana, CA, USA).

The particle number of mEVs solution were measured by nanoparticle tracking analysis and we monitored the body weight of mice every day, according to which we diluted the mEVs with PBS. The treatment groups were as follows: (1) DSS group: 12 mice were orally administered PBS for 30 days, followed by 3.5%DSS, and they continued to receive oral gavage of PBS for 7 days; (2) DSS + mEVs group: 12 mice were orally gavaged with mEVs (3.0 × 10^9^ particles per gram body weight) for 30 days, followed by 3.5%DSS, and they continued to receive oral gavage of mEVs 7 days. The dosage of mice consuming mEVs was approximately equivalent to a 62 kg adult human consuming 500 mL of milk [23]. Colon tissues were collected and stored at −80 °C for future analysis. The data of mice body weight, disease activity index and length of colon tissues were recorded in and presented in our previous study [24].

### 2.3. RNA Extraction and Construction of RNA Libraries

Total RNA containing miRNA was isolated from the colon tissues using the mirVana™ miRNA Isolation Kit (Thermo, Waltham, MA, USA). Briefly, the colon samples were first lysed in a denaturing lysis solution. Then the lysates were extracted with Acid-Phenol. Chloroform to remove the cellular components and further purified over glass-fiber filters by different procedures to yield total RNA and small RNAs for the follow-up sequencing. The RNA integrity was assessed by Agilent 2100 Bioanalyzer (Agilent, Santa Clara, CA, USA) with RNA integrity number ≥ 7. miRNA libraries were prepared by TruSeq Small RNA Sample Prep Kits (Illumina, San Diego, CA, USA). ncRNAs (lncRNAs and circRNAs) libraries were prepared by TruSeq Stranded Total RNA Prep Kit (Illumina, San Diego, CA, USA) following the manufacturer’s protocol. After library construction, the Agilent 2100 Bioanalyzer system was deployed to evaluate the quality. Then, the prepared libraries were sequenced on the Illumina Hiseq X Ten sequencing platform.

### 2.4. Bioinformatic Analysis of miRNAs

The length distribution of the clean reads in the *Mus musculus* genome was calculated. Rfam (v.10.1) and GenBank databases were used to align and identify small RNA classifications, including known miRNAs and unidentified miRNAs. The known miRNAs were identified against miRBase database (v. 22.0). miRDeep2 was used to predict previously unidentified miRNAs. The miRNAs expression was calculated by the transcripts per million method.

### 2.5. Bioinformatic Analysis of lncRNAs and circRNAs

The candidate lncRNAs transcripts were selected by Cuffcompare (v.2.2.1). The remaining transcripts were analyzed with CPC, CNCI, PFAM and PLEK to gain the lncRNA of Mus musculus. Gene quantitative analysis was performed using eXpress (v.1.5.1), and the FPKM mapped reads and counts value were gained. CIRI was used to scan for paired chiastic clipping signals, and circRNAs was further predicted according to junction reads and GT-AG cleavage signals. RPM reads was used to determine the level of circRNAs expression, and DESeq2 software (v1.50.2) was used for differential analysis.

### 2.6. Functional Enrichment Analysis

We performed GO enrichment and KEGG pathway analysis with the predicted target genes of DE lncRNAs. The source genes of DE circRNAs, and the predicted target genes of DE miRNAs were analyzed using R based on the hypergeometric distribution.

### 2.7. Construction of Competing Endogenous RNAs (ceRNAs) Network

The Miranda database was used to predict the regulatory relationships between the RNAs. Cystoscope (3.8.0, San Diego, CA, USA) was used to visualize the lncRNA-miRNA-mRNA and circRNA-miRNA-mRNA networks. Shapes and colors were used to imply different types of RNAs and regulatory relationships, respectively.

### 2.8. Quantitative Real-Time PCR Validation (qRT-PCR)

To measure the lncRNA and circRNA expression level, Evo M-MLV Plus 1st Strand cDNA Synthesis Kit (AG11615, Accurate Biotechnology, Guangzhou, China) and SYBR Green Premix Pro Taq HS qPCR Kit (AG11718, Accurate Biotechnology, China) were used for reverse transcription and qRT-PCR, respectively, following the manufacturer’s instructions. As for miRNAs, miRcute Plus MiRNA First-Strand cDNA Kit (AG11716, Accurate Biotechnology, China) and SYBR Green Premix Pro Taq HS qPCR Kit (AG11702, Accurate Biotechnology, China) were applied for qRT-PCR following the protocols. GAPDH was used as internal reference gene of mRNAs, lncRNAs and circRNAs, while U6 was considered as internal control for miRNAs. The relative gene expression was analyzed using the 2^−ΔΔCt^ method.

### 2.9. Statistical Analysis

The raw reads of miRNA libraries counted between 24.90 and 33.32 M, and the clean reads ranged from 24.02 to 24.89 M. The raw reads of ncRNAs (lncRNAs and circRNAs) libraries counted between 100.94 and 117.90 M, and the clean reads ranged from 98.75 to 115.46 M. The fold change (FC) of DE miRNAs, lncRNAs and circRNAs was calculated using the DESeq2 package in R [26]. DE RNAs were determined with both the *p* < 0.05 and FC > 1.5 or <0.67. qRT-PCR results were analyzed by *t*-test and presented as mean ± standard deviation. Normality and equality of variances were assessed using Shapiro–Wilk test and Levene’s test prior to *t*-test. *p* < 0.05 was considered statistically significant.

## 3. Results

### 3.1. Effects of mEVs Supplementation on the Expression of Colonic miRNAs

**Figure 1:**
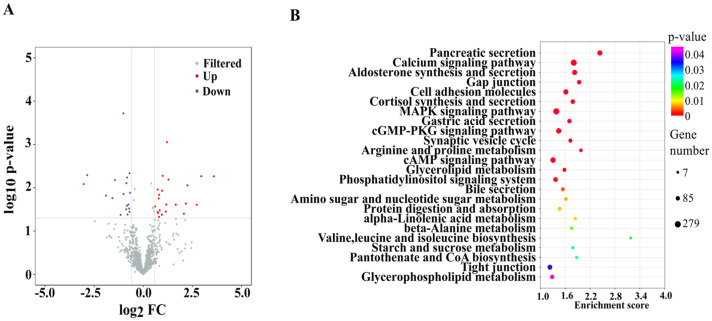
Oral mEVs changed the expression pattern of differentially expressed (DE) miRNAs in DSS-induced colitis (*n* = 6). (**A**) The volcano plots of DE miRNAs. (**B**) KEGG pathway analysis of target genes of DE miRNAs.

**Figure A2:**
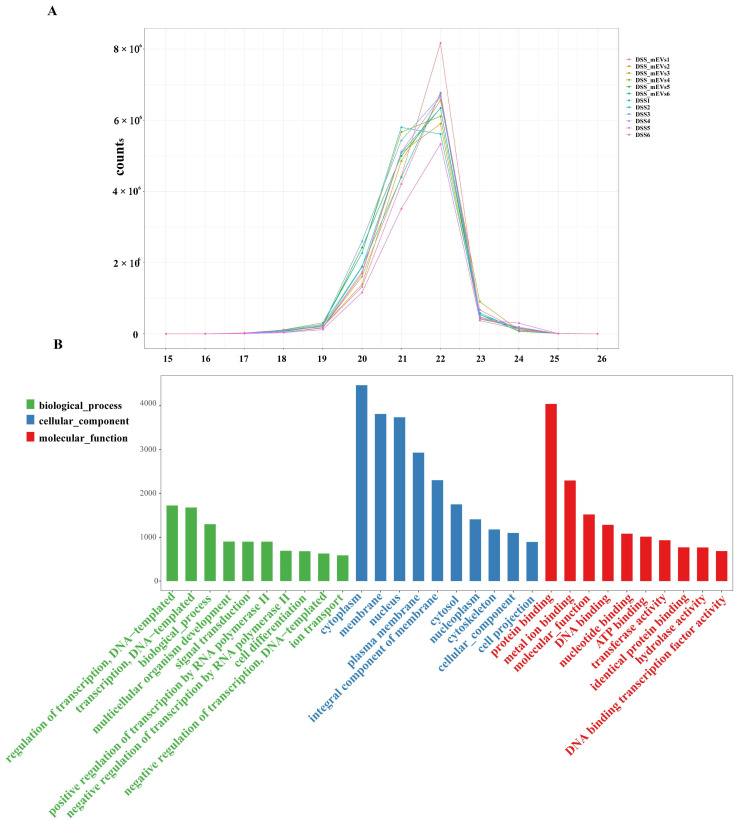
The length distribution of miRNAs in colonic tissues (**A**). GO enrichment analysis of target genes of differently expressed miRNAs (**B**).

**Figure A3:**
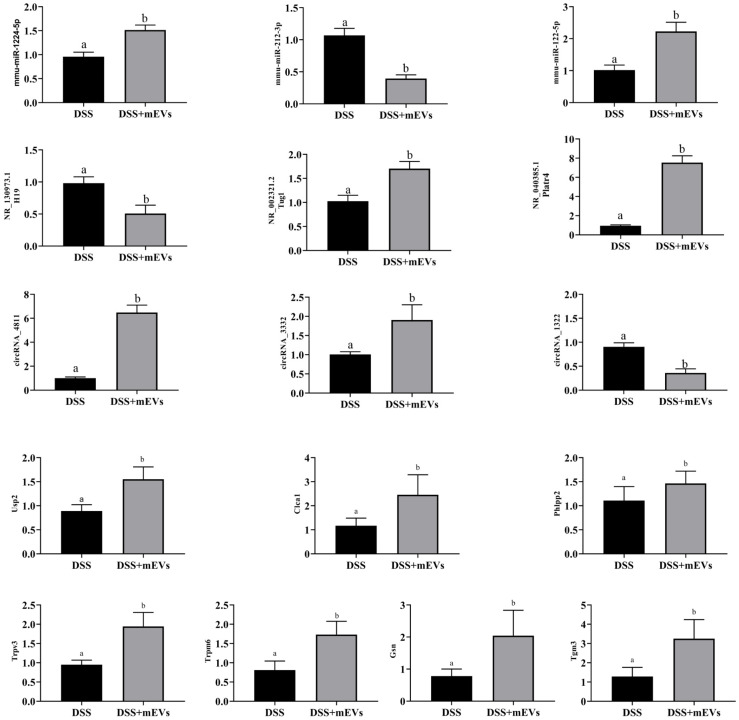
The relative expression of differently expressed miRNAs, lncRNAs, and circRNAs by qRT-PCR. Different letters in each figure panel indicate significant difference (*p* < 0.05).

The length distribution of miRNAs is presented in Figure A2, and the overwhelming length of miRNAs in all samples is 22 nt. A total of 40 significantly DE miRNAs were identified out of 761 types of miRNAs between the DSS and DSS + mEVs groups. mEVs significantly up-regulated 22 miRNAs (FC > 1.5 and *p* < 0.05) and down-regulated 18 miRNAs (FC < 0.67 and *p* < 0.05) (Figure 1A). The top five significantly up-regulated miRNAs were mmu-miR-5134-3p, mmu-miR-122-5p, mmu-miR-122b-3p, mmu-miR-669m-5p, mmu-let-7a-2-3p, while the significantly down-regulated miRNAs were mmu-miR-3112-5p, mmu-miR-6399, mmu-miR-6546-5p, mmu-miR-690 and mmu-miR-6539 in response to mEVs (*p* < 0.05) (Table 1). To further validate the miRNA sequencing result, qRT-PCR was used to detect the relative expression of three DE miRNAs (mmu-miR-1224-5p, mmu-miR-212-3p, and mmu-miR-122-5p). The results were consistent with the miRNA sequencing data (Figure A3).

We focused on the changes in GO terms and KEGG pathways related to the target genes of the DE miRNA. The top four most significantly enriched GO terms related to cell structure were cytoplasm (GO:0005737), protein binding (GO:0005515), membrane (GO:0016020) and nucleus (GO:0005634) (Figure A2). KEGG pathway enrichment analysis showed that mEVs supplementation affected mainly the amino acids metabolism, including valine, leucine and isoleucine biosynthesis (mmu00290), arginine and proline metabolism (mmu00330), beta-alanine metabolism (mmu00410), protein digestion and absorption (mmu04974), cysteine and methionine metabolism (mmu00270). Additionally, lipid metabolism was impacted, including pantothenate and CoA biosynthesis (mmu00770), alpha-linolenic acid metabolism (mmu00592), aldosterone synthesis and secretion (mmu04925), bile secretion (mmu04976), and glycerophospholipid metabolism (mmu00564) (Figure 1B).

### 3.2. Effects of mEVs Supplementation on the Expression of Colonic lncRNAs

**Figure 2:**
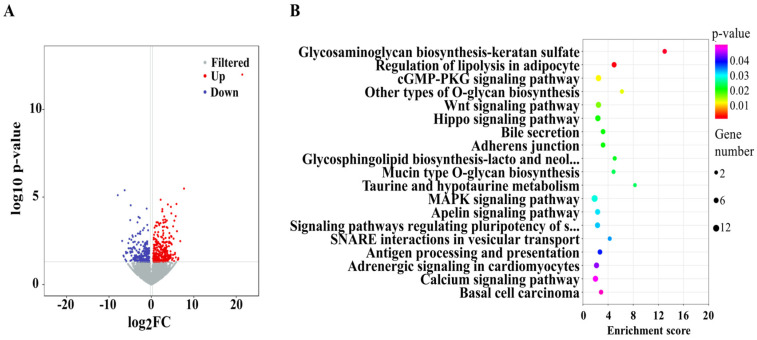
Oral mEVs changed the expression pattern of differentially expressed (DE) lncRNAs in DSS-induced colitis (*n* = 6). (**A**) The volcano plots of DE lncRNAs. (**B**) KEGG pathway analysis of target genes of DE lncRNAs.

According to the volcano plot, oral gavage of mEVs led to 314 significantly up-regulated lncRNAs and 227 significantly down-regulated lncRNAs (Figure 2A). In addition, mEVs also significantly increased the expression of lncRNA Platr4 and Tug1, and decreased the expression of lncRNA Snhg5, and H19. Table 2 presents a partial functional prediction of DE lncRNAs target genes, including the genes such as Klf4, Pax6, Ly86, Bhlhe40, Pla2g10, and Prdm1, which were tightly associated with colitis. To reliably validate the lncRNA sequencing result, we selected three DE lncRNAs (Tug1, H19, Platr4) for qRT-PCR analysis, which demonstrated the reliability of the sequencing results (Figure A3).

The GO analysis revealed that DE lncRNAs significantly enriched in the molecular function (such as protein binding, DNA binding transcription factor activity, and sequence-specific DNA binding), the cellular components (such as nuclear chromatin, cytoplasm, and ciliary rootlet), and the biological process (such as negative regulations of transcription by RNA polymerase II, DNA binding transcription factor activity, and neuron differentiation). The KEGG pathways were mainly enriched in nutrient metabolism and immune-related pathways. For example, glycosaminoglycan biosynthesis-keratan sulfate (mmu00533), regulation of lipolysis in adipocyte (mmu04923), mucin type O-glycan biosynthesis (mmu00512), and taurine and hypotaurine metabolism (mmu00430) were significantly annotated in metabolism. Wnt signaling pathway (mmu04310), Hippo signaling pathway (mmu04390), and MAPK signaling pathway(mmu04010) were also significantly enriched in response to mEVs (Figure 2B).

### 3.3. Effects of mEVs Supplementation on the Expression of Colonic circRNAs

**Figure 3:**
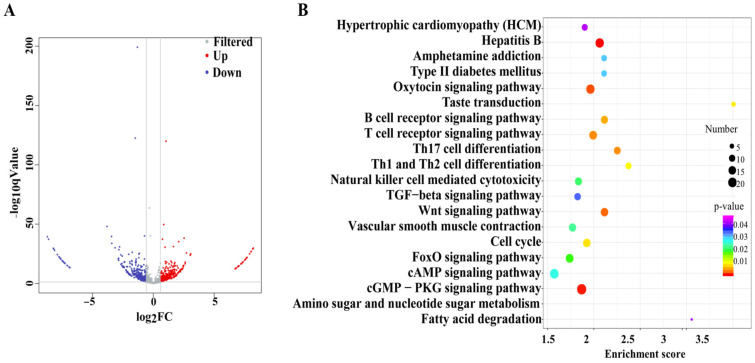
Oral mEVs changed the expression pattern of differentially expressed (DE) circRNAs in DSS-induced colitis (*n* = 6). (**A**) The volcano plots of DE circRNAs. (**B**) KEGG pathway analysis of target genes of DE circRNAs.

T A total of 643 DE circRNAs were identified, with 308 up-regulated and 335 down-regulated between the DSS and DSS + mEVs groups (Figure 3A). The top 10 significantly up-regulated and down-regulated circRNAs influenced by mEVs are presented in Table 3. qRT-PCR was used to measure the relative expression of three DE circRNAs (circRNA-3332, circRNA-4811, and circRNA-1322), which showed agreement with the sequencing data (Figure A3).

The biological processes of DE circRNAs were mostly enriched in positive regulation of transcription by RNA polymerase II (GO:0045944), regulation of chromosome segregation (GO:0051983), and regulation of transforming growth factor beta receptor signaling pathway (GO:0017015). The cellular components of DE circRNAs were mainly annotated in nuclear chromatin (GO:0000790), transcription factor complex (GO:0005667), RNA polymerase II transcription factor complex (GO:0090575). The molecular functions of these circRNAs were mostly associated with DNA binding transcription factor activity (GO:0003700), transcription regulatory region DNA binding (GO:0044212), and sequence-specific DNA binding (GO:0043565). Additionally, the top 20 KEGG pathways with the highest representation are presented in Figure 3B, with enrichments mainly related to immune system (T cell receptor signaling pathway, Th17 cell differentiation, B cell receptor signaling pathway, Th1 and Th2 cell differentiation, and natural killer cell mediated cytotoxicity) and nutrient metabolism (fatty acid degradation, and amino sugar and nucleotide sugar metabolism).

### 3.4. lncRNA-miRNA-mRNA Networks

**Figure 4:**
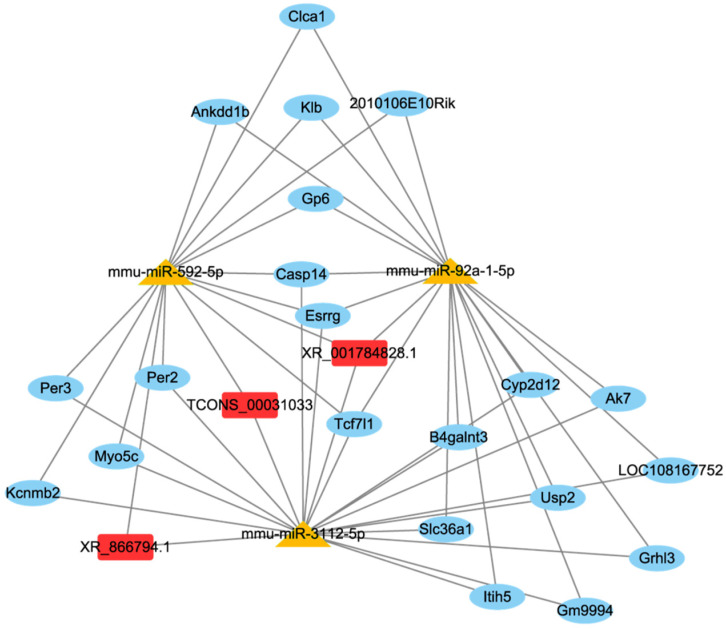
The ceRNA network of lncRNA-miRNA-mRNA.

lncRNA and circRNA have miRNA binding sites and could act as miRNAs sponge and indirectly modulating gene expression. This theory was referred to as the ceRNA network [27]. Hence, we constructed lncRNA/circRNA-miRNA-mRNA networks to illustrate the mechanisms by which ncRNA regulate gene expression. The results of DE mRNAs were presented in our previous study [24]. The network included 3 DE lncRNA, 3 DE miRNAs and 21 DE mRNAs (Figure 4). LncRNA XR_001784828.1 was predicted to bind to mmu-miR-92a-1-5p, mmu-miR-3112-5p, and mmu-miR-592-5p and further regulate Usp2 (ubiquitin specific protease 2), and Clca1 (chloride channel regulator, calcium-activated 1) expression. Both Usp2 and Clca1 were closely related to inflammatory response [28,29]. Therefore, the increase in lncRNA XR_001784828.1 induced by mEVs might regulate the inflammatory response through affecting the downstream target gene expression.

**Figure 5:**
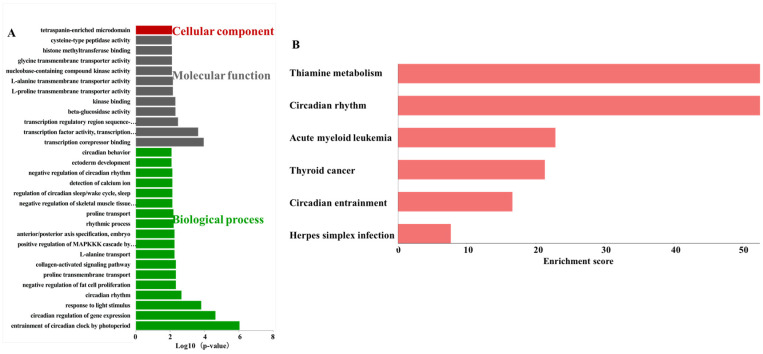
Enrichment analysis of lncRNA-miRNA-mRNA network. (**A**) The top 30 GO terms. (**B**) The significantly enriched KEGG pathways.

GO and KEGG enrichment analyses were used to predict the roles of DE lncRNAs in lncRNA-associated ceRNA networks. The top 30 significantly enriched GO terms consisted of 1 term in cellular component, 11 terms in molecular function and 18 terms in biological process (Figure 5A). Tetraspanin-enriched microdomain (GO:0097197) belonged to cellular component. The significantly enriched KEGG pathway included thiamine metabolism (mmu00730), circadian rhythm (mmu04710), acute myeloid leukemia (mmu05221), thyroid cancer (mmu05216), circadian entrainment (mmu04713), and herpes simplex infection (mmu05168) (Figure 5B).

### 3.5. circRNA-miRNA-mRNA Networks

**Figure 6:**
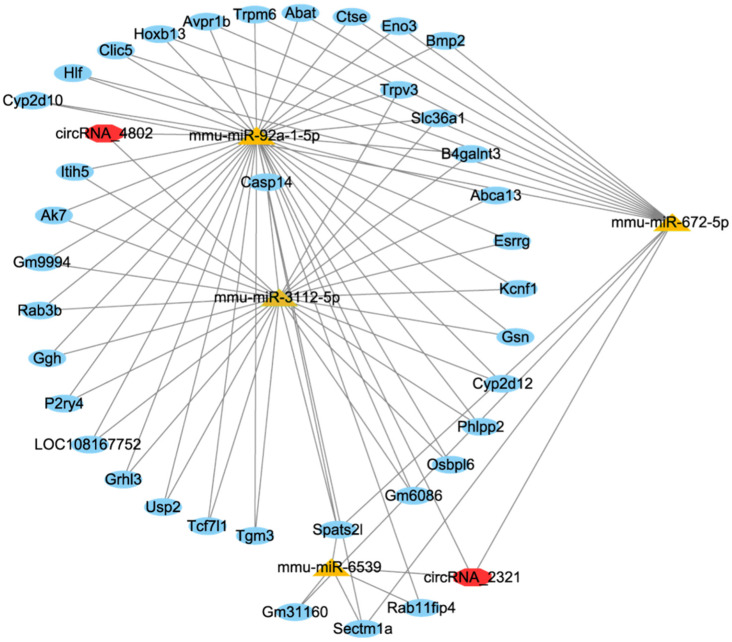
The ceRNA network of circRNA-miRNA-mRNA.

circRNA-miRNA-mRNA network consisted of 2 DE circRNAs, 4 DE miRNAs and 37 DE mRNAs (Figure 6). According to the circRNA-miRNA-mRNA network, circRNA_4802 was predicted to bind to mmu-miR-3112-5p and mmu-miR-92a-1-5p, further regulating the expression of Usp2, Gsn (gelsolin), Trpv3 (transient receptor potential vanilloid 3), Phlpp2 (pleckstrin homology domain leucine-rich repeat protein phosphatase 2), Tgm3 (transglutaminase 3), and Trpm6 (transient receptor potential melastatin 6).

**Figure 7:**
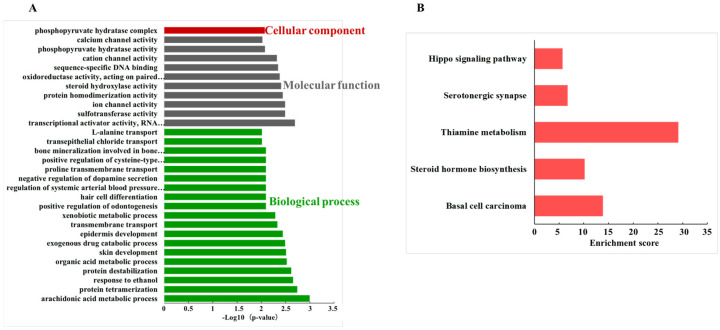
Enrichment analysis of circRNA-miRNA-mRNA network. (**A**) The top 30 GO terms. (**B**) The significantly enriched KEGG pathways.

GO enrichment analyzed the roles of circRNAs in circRNA-associated ceRNA networks. The top 30 significantly enriched GO terms included 1 term in cellular component, 10 terms in molecular function and 19 terms in biological process (Figure 7A). Phosphopyruvate hydratase complex (GO:0000015) was significantly enriched in cellular component. Meanwhile, the KEGG pathway for basal cell carcinoma (mmu05217), steroid hormone biosynthesis (mmu00140), thiamine metabolism (mmu00730), serotonergic synapse (mmu04726), and Hippo signaling pathway (mmu04390) were significantly influenced (Figure 7B).

## 4. Discussion

Recent studies revealed that ncRNAs were pivotal modulators of integral participants in the inflammatory disorders, which are involved in key colitis processes, such as immune cell activation, epithelial barrier integrity, and synthesis of pro-inflammatory mediators [30]. We have characterized the miRNA profiles of milk EVs in both Holstein and Swiss brown cattle, which contained large numbers of immune-related miRNAs [17,31,32]. Among the highly expressed miRNAs identified in bovine milk EVs, let-7a was found to overlap with the DE miRNAs in the colon tissue in this study. Therefore, we speculate that the observed up-regulated ncRNAs in colonic tissues might result from both direct transfer of cargo from milk EVs and indirect modulation of endogenous expression. The down-regulated ncRNAs were attributable to the endogenous regulation by milk EVs. Functional analysis of the DE ncRNAs in this study suggested mEVs might exert protective effects not only through modulation of inflammatory responses but also by enhancing ISCs function and epithelial regeneration.

In the case of inflammatory modulation, this study identified a large number of DE ncRNAs and related pathways. It was reported miR-147 exhibited potential anti-inflammatory properties because it could downregulate the expression of pro-inflammatory cytokines (TNF-α and IL-6) and negatively regulate TLR/NF-κB-mediated pro-inflammatory response [33]; miR-210 could modulate T cell differentiation by inhibiting HIF-1a expression, thereby alleviating intestinal inflammation [34]; the deficiency of miR-212 could improve the resistance to colitis, leading to increased populations of IL-10-producing cells [35]. Tug1 has been demonstrated to participate in the inflammatory activities in various occasions. Animal experiments have demonstrated that Tug1 overexpression could attenuate UC progression by regulating miR-142 and miR-29b-3p in the DSS-induced colitis in mice [36,37]. Platr4 is reported to inhibit NF-κB activity and deactivate Nlrp3 inflammasome by preventing binding of NF-κB to κB sites in the promoters of target genes, including Nlrp3 and Asc [38]. According to the ceRNAs network, the changed circRNA_4802 in this study could participate in the inflammatory response by binding to mmu-miR-3112-5p and mmu-miR-92a-1-5p, subsequently regulating the expression of Usp2, Gsn, Trpv3, and Phlpp2. Previous studies have found that Gsn could serve as a biomarker of inflammation to inhibit the secretion of pro-inflammatory cytokines of IL-6, TNF-α and NO by macrophages [39]. Trpv3 played an important role in maintaining the integrity of the epidermal barrier, whereas the level was usually decreased in ulcerative colitis [40]. Colonic Phlpp2 deficiency could directly activate the NF-κB signaling pathway, promoting colonic epithelial pyroptosis and aggravating the inflammatory response [41].

With respect to the enhance of ISCs function caused by the DE ncRNAs, Wnt and Hippo signaling pathways were primarily highlighted in this study. The expression of Tug1, Platr4, Snhg5, and H19 significantly changed after mEVs gavage, along with altered enrichment signaling pathways of Wnt and Hippo. Similarly, enrichment analysis of the circRNA-miRNA-mRNA network also focused on the Hippo signaling pathways. Leucine-rich repeat-containing G protein-coupled receptor 5 (LGR5) is a key marker and core functional molecular of ISCs, which could regulate their self-renewal, differentiation, and tissue regeneration [42,43]. It is reported that DSS-induced intestinal injury would deplete actively cycling LGR5^+^ ISCs and impair epithelial regeneration, which were regulated by the Wnt/β-Catenin signaling pathway [44,45]. Yes-associated protein (YAP) is a key downstream effector of the Hippo signaling pathway, which could promote the restoration of the intestinal mucosal barrier by regulating ISCs in IBD [46]. The Hippo signaling pathway was also reported to participate in the intestinal epithelial cell regeneration and barrier restoration in IBD [47]. Moreover, the DE circRNA of this study also enriched in Th17 cell differentiation. The Th17 cell-lineage-defining cytokine IL-17A could act on Lgr5^+^ ISCs to promote secretory cell lineage commitment and contribute to the integrity of the mucosa [48]. Therefore, the enrichment of DE ncRNAs in the Wnt and Hippo signaling pathways, as well as in Th17 cell proliferation, might account for the amelioration of colitis in this study.

Although we conducted a comprehensive transcriptomic analysis of the effects of mEVs on mice with colitis, including mRNA, miRNA, lncRNA, and circRNA profiling, along with their ceRNA Network, further in-depth functional characterization is warranted to elucidate their biological mechanism.

## Figures and Tables

**Table 1:** The top five significantly up-regulated and down-regulated miRNAs.

miRNAs	FC	log2FC	*p*-Value	Regulation	Length
mmu-miR-5134-3p	12.035	3.589	0.0054	Up	22
mmu-miR-122-5p	7.735	2.951	0.0054	Up	22
mmu-miR-122b-3p	6.603	2.723	0.0248	Up	22
mmu-miR-669m-5p	4.751	2.248	0.0088	Up	23
mmu-let-7a-2-3p	4.483	2.164	0.0233	Up	21
mmu-miR-6539	0.370	−1.435	0.0067	Down	24
mmu-miR-690	0.337	−1.568	0.0174	Down	22
mmu-miR-6546-5p	0.269	−1.894	0.0152	Down	24
mmu-miR-6399	0.139	−2.851	0.0051	Down	22
mmu-miR-3112-5p	0.123	−3.028	0.0083	Down	21

**Table 2:** The function prediction of DE lncRNAs target genes.

lncRNA ID	FC	log2FC	*p*-Value	lncRNA Symbol	Partner RNA Gene
XR_881290.2	37.713	5.237	0.0024	Gm12506	*Klf4*
ENSMUST00000123620	17.245	4.108	0.0209	ENSMUSG00000085375.1	*Klf4*
XR_380289.3	54.461	5.767	0.0283	Gm35028	*Prdm1*
ENSMUST00000215400	4.549	2.186	0.0000	ENSMUSG00000111078.1	*Prdm1*
NR_002867.2	6.044	2.596	0.0037	Pax6os1	*Pax6*
NR_117095.1	2.511	1.328	0.0483	Paupar	*Pax6*
ENSMUST00000135987	2.019	1.013	0.0185	ENSMUSG00000066176.3	*Klf4*
XR_390583.1	1.766	0.821	0.0223	Gm34343	*Klf4*
ENSMUST00000225533	0.257	−1.959	0.0354	ENSMUSG00000114249.1	*Ly86*
XR_001780900.1	0.127	−2.980	0.0038	Gm29590	*Ly86*
NR_040757.1	0.263	−1.928	0.0478	0610040F04Rik	*Bhlhe40*
ENSMUST00000204759	0.039	−4.667	0.0403	ENSMUSG00000087341.4	*Bhlhe40*
NR_040574.1	1.843	0.882	0.0194	Pla2g10os	*Pla2g10*
ENSMUST00000229709	0.036	−4.809	0.0085	ENSMUSG00000115907.1	*Pla2g10*

**Table 3:** The top 10 significantly up-regulated and down-regulated circRNAs.

circRNA	FC	log2FC	*p*-Value	Regulation
circRNA_3372	293.226	8.196	3.12 × 10^−32^	Up
circRNA_0047	285.584	8.158	1.49 × 10^−31^	Up
circRNA_2322	260.742	8.026	2.60 × 10^−29^	Up
circRNA_4911	258.066	8.012	4.56 × 10^−29^	Up
circRNA_3454	238.264	7.896	3.04 × 10^−27^	Up
circRNA_0429	205.438	7.683	3.83 × 10^−24^	Up
circRNA_1636	203.277	7.667	6.18 × 10^−24^	Up
circRNA_3275	201.976	7.658	8.25 × 10^−24^	Up
circRNA_0532	199.907	7.643	1.31 × 10^−23^	Up
circRNA_0375	189.564	7.567	1.32 × 10^−22^	Up
circRNA_2713	0.005	−7.657	2.58 × 10^−23^	Down
circRNA_1409	0.005	−7.720	3.63 × 10^−24^	Down
circRNA_0522	0.005	−7.759	1.06 × 10^−24^	Down
circRNA_2777	0.004	−7.884	1.66 × 10^−26^	Down
circRNA_3637	0.004	−7.892	1.28 × 10^−26^	Down
circRNA_4845	0.004	−8.121	2.78 × 10^−30^	Down
circRNA_5262	0.003	−8.188	1.95 × 10^−31^	Down
circRNA_4339	0.003	−8.227	4.04 × 10^−32^	Down
circRNA_3379	0.003	−8.616	6.85 × 10^−40^	Down
circRNA_3233	0.002	−8.714	4.25 × 10^−42^	Down

## Data Availability

All the raw sequences of the transcriptome can be found in the NCBI Sequence Read Archive (SRA) under accession number PRJNA821425.

## Abbreviations

The following abbreviations are used in this manuscript:

ceRNAs	competing endogenous RNAs
circRNAs	circular RNAs
DE	differentially expressed
DSS	dextran sulfate sodium
EVs	extracellular vesicles
FC	fold change
GAPDH	Glyceraldehyde-3-phosphate dehydrogenase
GO	Gene Ontology
HSP70	Heat Shock Protein 70
IBD	Inflammatory bowel diseases
IL-17A	interleukin-17A
ISCs	intestinal stem cells
KEGG	Kyoto Encyclopedia of Genes and Genomes
LGR5	leucine-rich repeat-containing G-protein-coupled receptor 5
lncRNA	long non-coding RNA
MAPK	Mitogen-Activated Protein Kinase
mEVs	Milk-derived extracellular vesicles
NLRP3	NLR family pyrin domain containing 3
ncRNAs	non-coding RNAs
NTA	nanoparticle tracking analysis
TEM	transmission electron microscopy
Th17	T helper 17 cell
TSG101	Tumor susceptibility fene 101
YAP	Yes-associated protein
